# Modeling cheatgrass distribution, abundance, and response to climate change as a function of soil microclimate

**DOI:** 10.1002/eap.3028

**Published:** 2024-09-16

**Authors:** Tyson J. Terry, Stuart P. Hardegree, Peter B. Adler

**Affiliations:** ^1^ Department of Wildland Resources and the Ecology Center Utah State University Logan Utah USA; ^2^ Department of Disturbance Ecology and Vegetation Dynamics Bayreuth University Bayreuth Bavaria Germany; ^3^ USDA‐ARS Northwest Watershed Research Center Boise Idaho USA

**Keywords:** annual grass, biological invasion, *Bromus tectorum*, germination, rate sum, resistance and resilience, SHAW model

## Abstract

Exotic annual grass invasions in water‐limited systems cause degradation of native plant and animal communities and increased fire risk. The life history of invasive annual grasses allows for high sensitivity to interannual variability in weather. Current distribution and abundance models derived from remote sensing, however, provide only a coarse understanding of how species respond to weather, making it difficult to anticipate how climate change will affect vulnerability to invasion. Here, we derived germination covariates (rate sums) from mechanistic germination and soil microclimate models to quantify the favorability of soil microclimate for cheatgrass (*Bromus tectorum* L.) establishment and growth across 30 years at 2662 sites across the sagebrush steppe system in the western United States. Our approach, using four bioclimatic covariates alone, predicted cheatgrass distribution with accuracy comparable to previous models fit using many years of remotely‐sensed imagery. Accuracy metrics from our out‐of‐sample testing dataset indicate that our model predicted distribution well (72% overall accuracy) but explained patterns of abundance poorly (*R* ^2^ = 0.22). Climatic suitability for cheatgrass presence depended on both spatial (mean) and temporal (annual anomaly) variation of fall and spring rate sums. Sites that on average have warm and wet fall soils and warm and wet spring soils (high rate sums during these periods) were predicted to have a high abundance of cheatgrass. Interannual variation in fall soil conditions had a greater impact on cheatgrass presence and abundance than spring conditions. Our model predicts that climate change has already affected cheatgrass distribution with suitable microclimatic conditions expanding 10%–17% from 1989 to 2019 across all aspects at low‐ to mid‐elevation sites, while high‐ elevation sites (>2100 m) remain unfavorable for cheatgrass due to cold spring and fall soils.

## INTRODUCTION

Invasive annual grasses have been linked to a worldwide decline of biodiversity and ecosystem functioning in water‐limited systems (D'Antonio & Vitousek, 1992; Davies, 2011; Ostoja & Schupp, 2009). Once present, these grasses accelerate fire return intervals (Fusco et al., 2019), increase nitrogen storage in soils (Wolkovich et al., 2010), and compete with native vegetation for soil moisture (Melgoza et al., 1990). Positive feedback loops with fire and nutrient cycles have enabled invasive annual grasses to dominate substantial portions of water‐limited systems that now burn two to four times more frequently than native communities (Bradley et al., 2018; D'Antonio & Vitousek, 1992; Fusco et al., 2019; Pastick et al., 2021). Information about the current and future distribution of these grasses is crucial to guide management decisions and wildfire planning (Chambers, Bradley, et al., 2014; Chambers, Miller, et al., 2014).

Climate change will likely alter the vulnerability of different ecosystems to annual grass invasion and dominance (Catford et al., 2019). In the sagebrush steppe, arid conditions are becoming more prevalent (Ficklin & Novick, 2017) due to increases in annual minimum temperature, increased climatic water deficit, and less summer rainfall (Smith et al., 2022). These temporal trends have the potential to impact invasion, as colder and wetter locations that have previously been classified as resistant to invasion (Chambers, Bradley, et al., 2014; Chambers, Miller, et al., 2014) are now becoming warmer and drier (Bradford et al., 2019; Smith et al., 2022). Despite many experimental and observational studies, there remains uncertainty surrounding future effects of climate change on cheatgrass (*Bromus tectorum* L.) distribution. Large‐scale studies predict range expansion of annual grasses (Pastick et al., 2021; Smith et al., 2022), but results of cheatgrass‐specific studies vary. While some predict increases in abundance (Boyte et al., 2016), others predict no changes (Brummer et al., 2016; Zimmer et al., 2021) or a dependence on future precipitation scenarios (Bradley, 2009). Experimental studies show both gains in fitness with warming (Blumenthal et al., 2016; Compagnoni & Adler, 2014) and no effect (Larson et al., 2017, 2018; Zelikova et al., 2013). The signal may be unclear because of site‐level factors that influence how atmospheric weather impacts soil temperature and moisture. What is clear is a lack of consensus on how changing climate will facilitate or inhibit cheatgrass expansion across western North America.

Our understanding of the climatic niche of invasive annual grass species and potential shifts in distribution due to climate change could be improved by new modeling approaches that directly link field observations with soil microclimate (Bradford et al., 2019: Boehm et al., 2021; Hardegree et al., 2022). Current landscape‐scale mapping approaches use reflectance indices such as normalized difference vegetation index (NDVI) to estimate the distribution and abundance of invasive annual grasses (Bradley et al., 2018; Downs et al., 2016; Pastick et al., 2021). While these models are useful for mapping large areas and make it possible to study the distribution or abundance of invasive annual grass species without extensive field sampling, they do not directly describe species–climate relationships. Rather, one model links cheatgrass abundance or distribution with reflectance, and a second analysis correlates climate variables with estimates of abundance or distribution. Quantifying error propagation from the first model through to the second is challenging; ignoring that error means overestimating the certainty of the species–climate correlations. Previous studies linking climate to large‐scale cheatgrass distribution (Bradley, 2009; Boyte et al., 2016) correlated climate metrics to remotely sensed estimates of cheatgrass distribution that either included large uncertainty (*R* ^2^ = 0.21) (Peterson, 2006) or required several years of imagery to distinguish between cheatgrass‐dominated sites and sites dominated by other vegetation types (Bradley & Mustard, 2005). An approach that directly explains spatially and temporally extensive field observations with fine‐scale climate indices would improve inference about species–climate relationships and could also account for interannual variation in abundance.

Persistent cheatgrass presence and abundance is largely driven by soil moisture and temperature (Chambers et al., 2007; Roundy et al., 2018), but regional‐scale studies are generally constrained to using coarse climate data from gridded climate products. These products provide information about precipitation and temperature at 0.8–4 km^2^ spatial resolution (Abatzoglou, 2013; Daly et al., 2008), but do not capture the shifts in soil moisture and temperature that occur with finer scale variation in topography and soil type (Hardegree et al., 2022) and which determine local cheatgrass dynamics (Bishop et al., 2019; Condon et al., 2011; Roundy et al., 2007). Soil moisture models use edaphic characteristics and topography, in addition to weather inputs, to explicitly account for factors that modify soil microclimate (Hardegree et al., 2022) and calculate soil moisture and temperature estimates at a smaller scale (10 m^2^ with current methods).

By combining germination models with output from a soil microclimate model, we can link species‐specific physiology with fine‐scale information about soil temperature and moisture across large spatial extents (Terry et al., 2022). Hydrothermal and thermal‐germination models have previously been used to predict cheatgrass response to microclimate variability at small scales (Hardegree et al., 2017, 2018; Rawlins et al., 2012; Roundy et al., 2007). These models not only predict the timing of cumulative germination response in the seedbed but also yield rate sum metrics that can be used to quantify the favorability of soil microclimate for plant establishment and growth (Hardegree et al., 2020). Rate sum metrics account for cumulative effects of small disparate windows of germination and growth favorability that are exploited by annual plants (Terry et al., 2022). Rate sum values have been shown to capture shifts in soil microclimate that occur with gradients of elevation and topography, which are generally associated with ecological resilience and resistance to cheatgrass invasion (Chambers, Bradley, et al., 2014; Chambers, Miller, et al., 2014; Hardegree et al., 2022; Roundy et al., 2018).

The objective of this study was to combine a mechanistic understanding of cheatgrass germination with soil microclimate data to predict its abundance and distribution across the sagebrush steppe. Specifically, we asked, (1) can we accurately model cheatgrass distribution and abundance using solely germination metrics of soil microclimate favorability? And, (2) have microclimatic conditions become more favorable for cheatgrass over the last 30 years in the sagebrush steppe?

## METHODS

### Overview of approach

**FIGURE 1:**
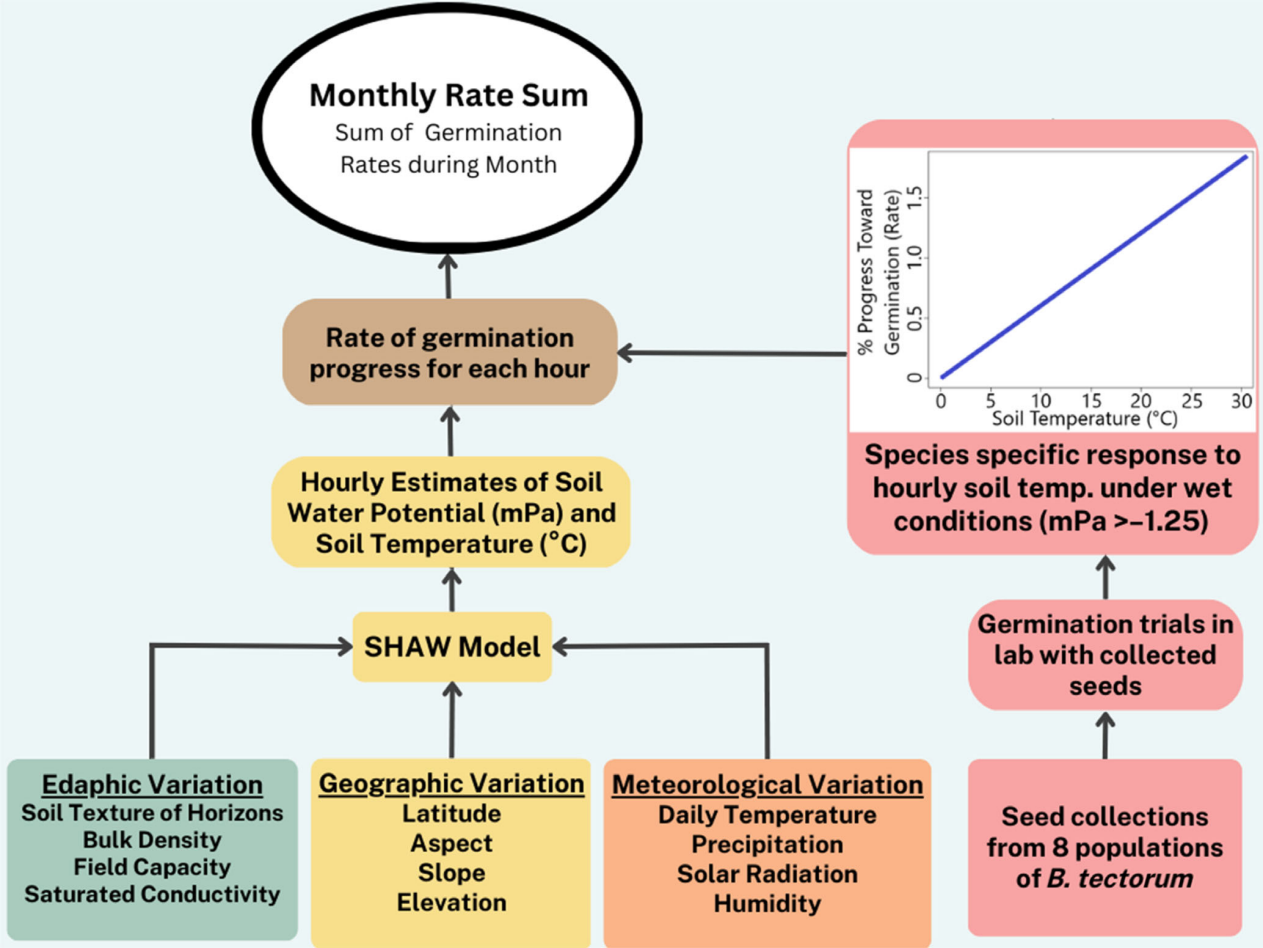
Flowchart indicating inputs used to estimate soil microclimate conditions and how they are combined with germination models to produce monthly rate sum values. SHAW, Simultaneous Heat and Water.

We used a species‐specific germination model for cheatgrass to quantify the favorability of soil microclimate for growth as a function of soil temperature and moisture estimates in the sagebrush steppe system in the western United States. We use hourly rates of germination progress for cheatgrass and sum them over time to capture the favorability of soils for germination and growth (Figure 1). We used monthly germination rate sums (Hardegree et al., 2020) to explain field observations of cheatgrass presence (>2% cover) and abundance. We split the dataset into a training and out‐of‐sample (OOS) testing dataset, with both spanning the temporal (2002–2016) and spatial range of the dataset to test model performance. We created two models, one to predict presence/absence and one to predict abundance. After testing both models on the OOS dataset, we applied the model to simulated soil conditions at all sites (training and testing) from 1989 to 2019 and analyzed trends in potential distribution and abundance across all sites.

### Soil water model

We used the Simultaneous Heat and Water (SHAW) Model (Flerchinger et al., 2012) to generate soil moisture and soil temperature estimates for each site. This model uses atmospheric, edaphic, and geographic variables to model soil water and temperature as a function of soil depth (Figure 1). Soil texture data for each site were acquired from OpenLandMap (Hengl, 2018) for the three soil depths (0–10, 10–30, and 30–60) for each simulated field site. Other edaphic variables (field capacity, bulk density, and saturated conductivity) needed for the SHAW model parameterization were calculated using soil texture via Saxton equations (Saxton et al., 1986). Daily temperature, precipitation, solar radiation, relative humidity, and wind variables were obtained from the gridMET gridded historical climate database (4000 m spatial resolution, daily temporal resolution) (Abatzoglou, 2013). The geographic inputs of aspect, slope, and elevation were derived from topographic data (10 m spatial resolution) acquired from Farr et al. (2007). We did not include optional vegetation inputs in the model for simplicity and for potential application to post‐wildfire landscapes with minimal vegetation. From this model, we generated hourly estimates of soil temperature (in degrees Celsius) and soil water potential (in megapascals) at 2 cm depth, a depth common for seeding success in restoration settings. These estimates from the SHAW model were then used as input into the wet‐thermal germination models to estimate hourly and cumulative rate sum values.

### Germination model

Our approach utilizes germination curves that specify how hourly germination rate changes with temperature when soil is wet (>−1.25 MPa). With soil moisture and temperature metrics as inputs, we calculate the sum of germination rates for the early spring and late fall months. We used germination models with soil conditions at 2 cm depth to provide hourly rate sum metrics throughout the year at all sites. In this study, we used the rate sum of the 35% subpopulation of seeds for our analysis (Terry et al., 2022). This metric explicitly represents the number of sequential times during a given time period where conditions were sufficient for 35% of a given seed population to germinate (Hardegree et al., 2020). The rate sum value for a given time period is a quantitative index of seedbed favorability for germination and growth (Hardegree et al., 2013; Terry et al., 2022).

Germination rates and rate sum calculations were based on wet‐thermal germination models (Rawlins et al., 2012; Roundy et al., 2007), an approach that calculates germination rate, or the relative progress toward germination during a specific time period, according to soil temperature under continuously wet conditions (soil water availability <−1.25 MPa). Methods for predicting germination response to temperature above threshold levels of soil water availability have been previously described and validated (Hardegree et al., 2018; Roundy et al., 2007). We used data from previous germination trials of cheatgrass seeds collected from eight different field sites in the sagebrush steppe (Hardegree et al., 2010; Roundy et al., 2007). We averaged germination curves that explain how hourly progress toward germination varies under wet condition under different temperatures. This average was done across all cheatgrass collection sites (seedlots) for the 35% subpopulation to produce Equation (1). We chose the 35% subpopulation, or germination rates for 35% of the seeds to germinate, as this grouping captures the majority of high‐quality seed with the best chance for growth and fecundity (Baskin & Baskin, 2014). Equation (1) was used to calculate the germination rate of cheatgrass for each hour (*t*) as a function of soil temperature (*T*) when soil temperature is greater than 0°C and soil water potential is greater than −1.25 MPa (Roundy et al., 2007; Terry et al., 2022). Outside these conditions, germination rate was considered zero.

(1) $$\begin{array}{l} {\text{Germination Rate}}_{t}=\\ \left\{\begin{array}{ll} \;1.29\times 10^{-4}+T_{t}\times -1.25\times 10^{-5}+T_{t}\times 6.16\times 10^{-4}, & \;T>0^{{}^{\circ}}C\;\text{and}\;\mathrm{MPa}>-1.25\\ \;0\;, & \;\text{otherwise}. \end{array}\right. \end{array}$$

### Cheatgrass presence and abundance data

**FIGURE 2:**
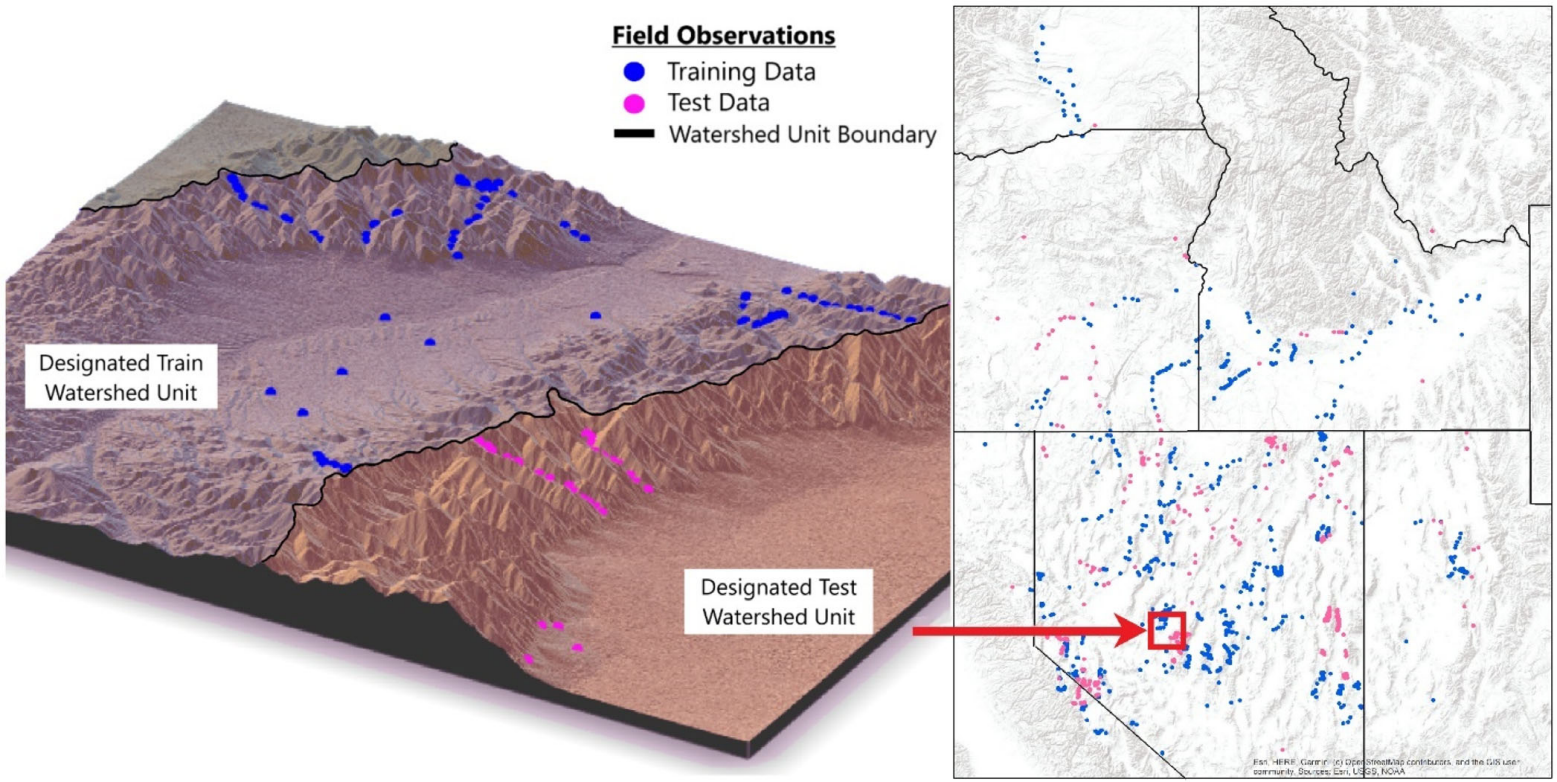
Location of all field observations (right) and an example of how watershed units were used to split the data into training (blue points) and testing (pink) sets. This approach allowed us to test our model on a dataset that matches the spatiotemporal extent of the training dataset while ensuring a degree of independence between the two datasets.

We used field observations of cheatgrass presence/absence and abundance from 2662 field observations collected from 2002 to 2016 (Appendix S1: Table S1), which was a subset of data compiled by Bradley et al. (2018). The field observations span much of the sagebrush steppe in the western United States, with sites in Idaho, Utah, Nevada, California, Oregon, and Washington (Figure 2). Our dataset does not include any observations in the Mojave Desert or eastern portions of the sagebrush steppe in Wyoming, Montana, or Colorado. Most of the data was collected using line transects, with some of the cover estimates coming from ocular estimates and quadrat frames. We analyzed a subset of the data consisting of all sites with measures of absolute cover (area covered by species/total geographic area) rather than relative cover (% of total vegetative cover) to train and test our model to predict estimates of cheatgrass cover.

### Model training and testing

We split our data into a training set and an OOS testing set to validate the accuracy of our model. We utilized eight‐digit watershed units (USGS), a spatial delineation that identifies watershed basins, to identify independent spatial groupings of field observations (Figure 2). Altogether our data encompass 102 unique watershed units within the sagebrush steppe. Field observations were randomly separated into testing (1/3 of watershed units and 29% of data) and training data (2/3 of watershed units and 71% data). This resulted in a training dataset composed of 884 presence points and 785 absence points and an independent testing dataset composed of 533 presence points and 449 absence points. Both the training and test datasets comprise field observations spanning the temporal and spatial range of our dataset (Figure 2), with no repeat observations. Histograms of percent cheatgrass cover were generated to ensure similar distribution of cheatgrass cover between training and testing dataset (Appendix S1: Figure S1).

We assessed prediction accuracy based on the model's ability to predict presence (>2% cheatgrass cover) and percent cover at OOS test sites in the specific year of the field observation. Accuracy metrics are percentage of test sites correctly identified as present/absent and *R* ^2^ value for cover predictions across OOS test sites (observed vs. predicted). We chose this as our comparison metric for cover predictions to allow comparison with models from previous studies, which do not always report other metrics such as mean absolute error or root mean square error.

### Cheatgrass model

We used a generalized additive model (GAM) from the mgcv package (Wood, 2004) in R (R Core Team, 2019, version 3.6.1) to relate spatial and temporal soil favorability metrics to cheatgrass presence and cheatgrass abundance. We selected a GAM modeling approach to account for nonlinear effects of microclimate that would require complex interaction terms in a linear model. Our response variables were distribution (presence/absence) and abundance (cheatgrass cover), which we analyzed in two separate models. We chose a cubic‐splines smoothing approach to allow knots to spread evenly throughout covariate values (Wood, 2006) to ensure all combinations of covariate values were considered within the smoothing terms of the model.

Our distribution model was a GAM binomial model, with field observations of cheatgrass cover >2% considered as species presence. Our abundance model was a GAM model with a normal error distribution. We used four covariates that represent both temporal and spatial variation of microclimate in the form of rate sum. Spatial covariates were mean rate sum values of spring (March) and late fall (October–December) and were scaled spatially by subtracting off the mean of all sites and dividing by the SD of all sites for each variable, such that a site with a value of 0 would indicate an average value relative to all the other sites. The spatial covariates describe variation in climate among locations. Temporal covariates were rate sum values of spring (March) and late fall (October–December) soil conditions immediately preceding the field observation. These measurements were scaled temporally (across years at each site) by subtracting off the site‐level mean and dividing by site‐level SD of each variable such that a value of 0 would indicate average conditions within a given site. These temporal covariates describe interannual variation in weather for each location.

To select the four covariates described in the previous paragraph, we first computed correlations of cheatgrass cover in our training dataset with all individual monthly rate sums and groupings of monthly values to seasonal sums that previous studies suggested may influence cheatgrass abundance and distribution (Bradley et al., 2016; Roundy et al., 2018). After creating models with the top 10 most correlative (with cheatgrass cover) rate sum metrics, we found that having many nonlinear parameters did not facilitate interpretation, and that by reducing the covariates to the two most correlated rate sum values of late fall (October–December) and Spring (March), we could retain most of the predictive power of the models while increasing interpretability. We assumed that susceptibility to invasion was driven by both spatial and temporal microclimate dynamics and thus included both the spatial and temporal values of these metrics to allow interannual variation and average soil microclimate conditions to inform our models.

Models were checked for goodness‐of‐fit on the training data using the gam.check function in the mgcv package (Wood, 2005). Specifically, we checked the basis dimensions of smoothing terms to ensure that they were not so small that they force over‐smoothing and checked residuals for over‐dispersion. While we monitored specific GAM model diagnostics, we placed more emphasis on model validation with our OOS testing dataset.

## RESULTS

### Model performance

**FIGURE 3:**
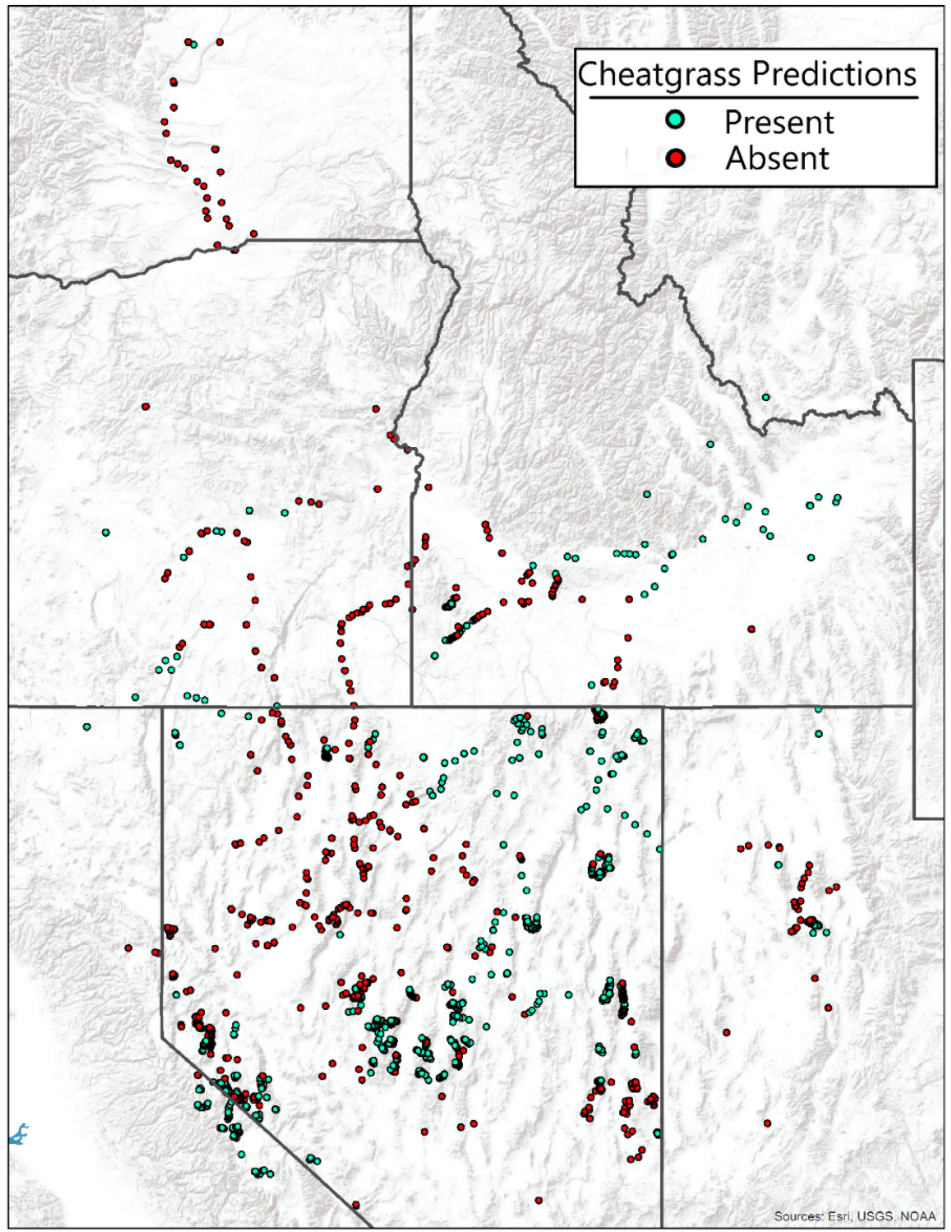
Map of model predictions for cheatgrass presence/absence using our final model at both testing and training sites.

**FIGURE 4:**
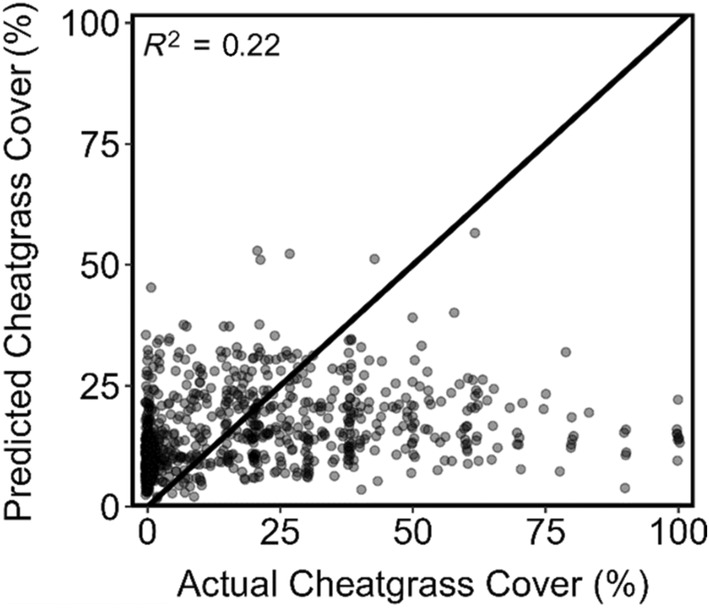
Predictions of cheatgrass cover versus actual values on independent (out‐of‐sample) test dataset. Line represents 1:1 ratio.

All spatial and temporal parameters within our model had significant effects (*p* < 0.001, Appendix S1: Tables S1 and S2) on cheatgrass distribution (presence/absence) and abundance (cheatgrass cover). Accuracy metrics for the OOS testing dataset show that our mechanistic soil favorability metric covariates predicted presences with 77.8% accuracy and absences with 65.6% accuracy, with an overall balanced accuracy of 71.7% (Figure 3). Soil favorability covariates predicted abundance less well (*R* ^2^ 0.22 testing, 0.22 training), with poor ability to distinguish high and low cover sites (Figure 4).

### Response to spatial variation in microclimate

**FIGURE 5:**
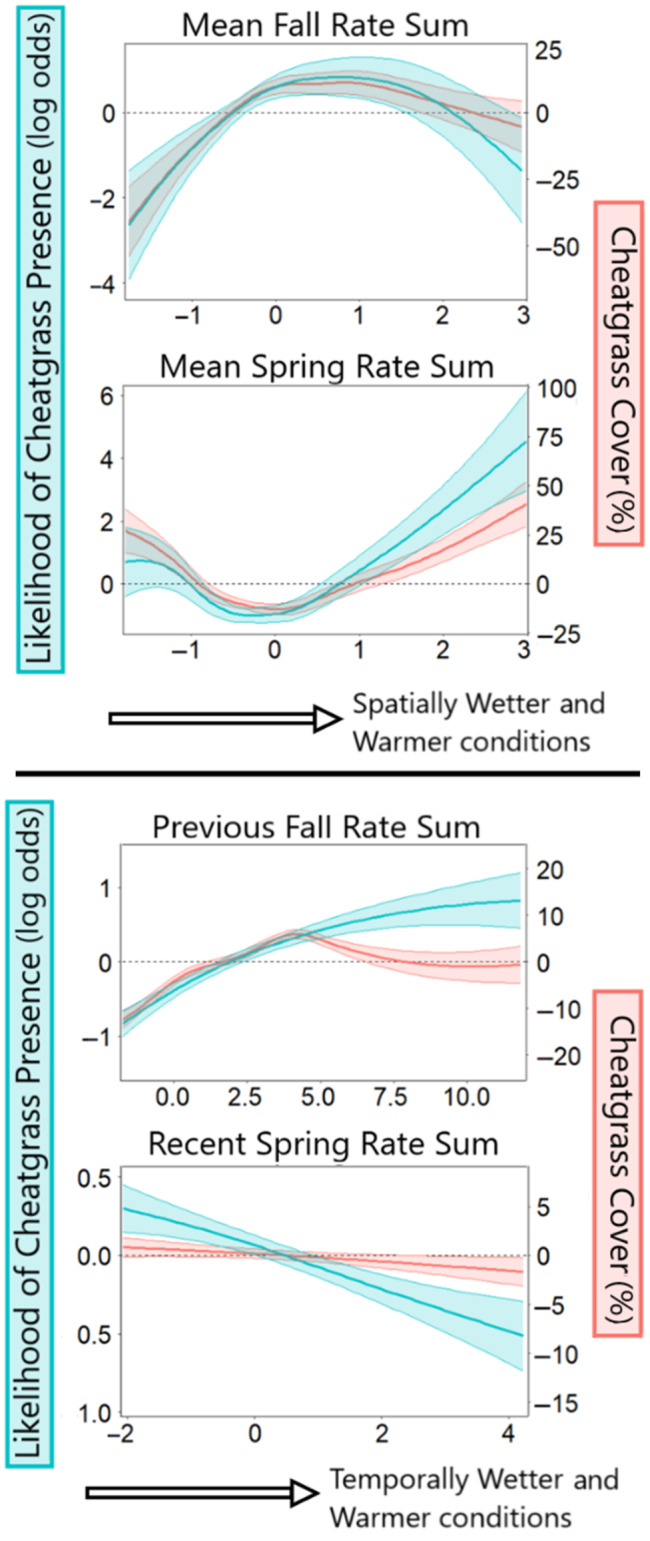
Plots showing smoothed parameter effects. *Y*‐axis values indicate magnitude of the effect, and *X*‐axis values indicate possible parameter values within the dataset. Blue values represent the likelihood (log odds) of cheatgrass presence at a site. Red values indicate impacts on cheatgrass cover. Top panel includes average rate sum values that were scaled spatially with other sites in the dataset. Bottom panel includes recent rate sum values corresponding to the annual conditions during the year of observation. Recent rate sum values were scaled within site to indicate deviations from a site's respective mean.

Cheatgrass presence and cover responded in similar ways to spatial variation in soil microclimate (Figure 5). GAM coefficient curves indicate that cheatgrass performs best in locations with warmer and wetter soils in spring and fall. Cheatgrass abundance and distribution (persistence) responded to average fall rate sum with a concave down shape, indicating a preference for locations that on average have moderately wetter and warmer fall soils. Abundance and distribution responded to spring rate sum values with a concave down but a generally positive slope, indicating a preference for locations that on average have warmer and wetter spring soils (Figure 5).

### Response to temporal variation in microclimate

Temporal variation in fall and spring soil conditions had significant effects (*p* < 0.001) on interannual cheatgrass distribution and abundance (Figure 5). Wetter and warmer soils from the previous fall increased the likelihood of presence but the benefits of warm and wet fall soils for cover declined at high values, exhibiting a concave‐down shape with optimal conditions occurring in slightly above average years. Years with wetter and warmer spring soils were associated with lower cover and probability of abundance (Figure 5).

### Long‐term trends

**FIGURE 6:**
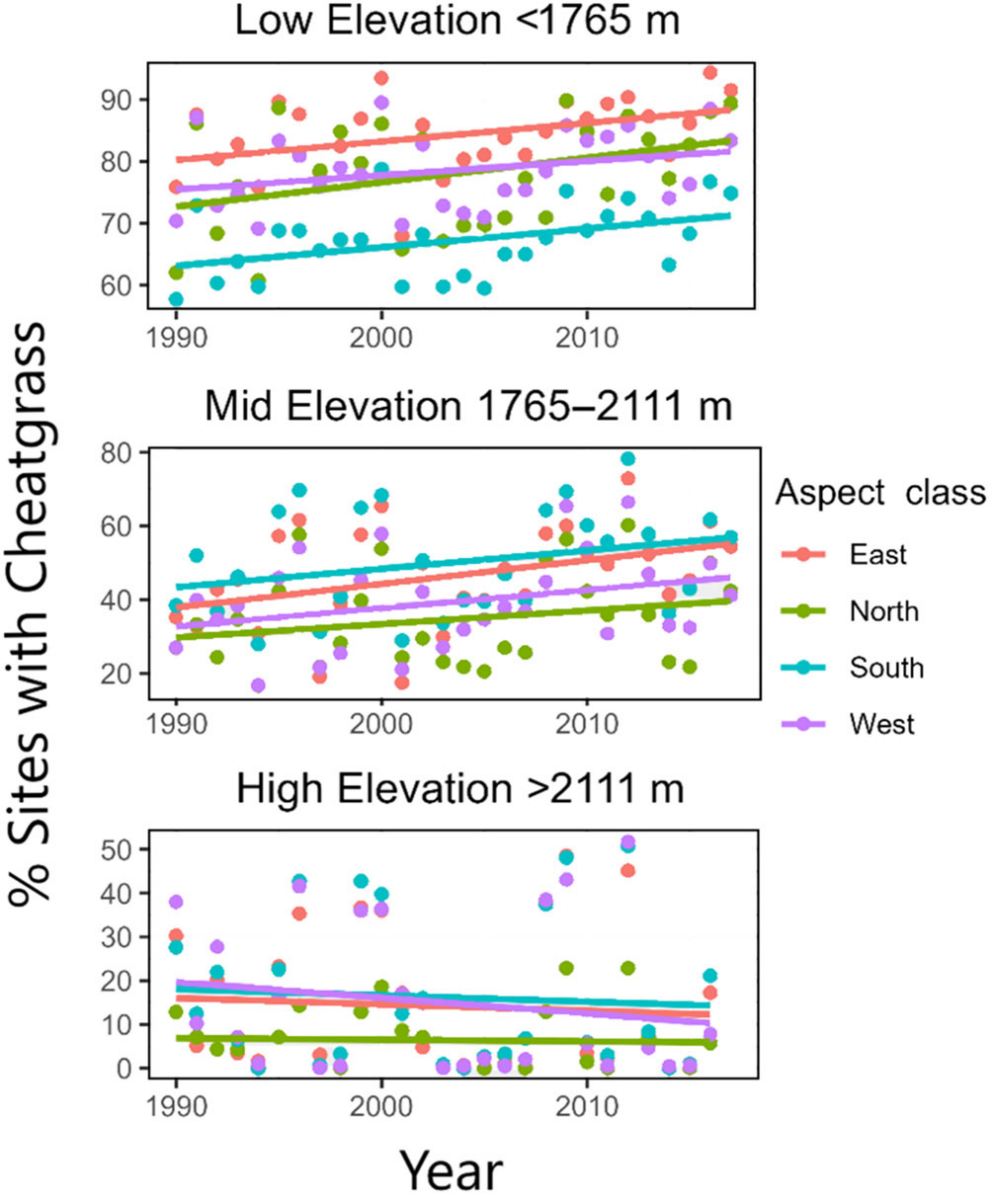
Predicted distribution of cheatgrass across sites according to elevation and aspect. Each dot represents the percent of total sites predicted to be occupied by cheatgrass in each year with color indicating different aspects. The elevation categories represent the bottom, middle, and top tercile of the dataset.

Given the reasonable predictive ability of our distribution (presence) model for our OOS testing dataset, we applied our model to soil metrics at all sites (*n* = 2662) across the years 1990–2019 to hindcast trends in cheatgrass distribution. We found that the conditions identified in our model as favorable for cheatgrass presence are becoming more prevalent across our study sites (Figure 6) during the period 1990–2019. Specifically, we saw evidence of climate change‐induced range expansion, with predicted cheatgrass presence expanding 10%–17% across our mid‐ and low‐elevation sites (Figure 6). Higher elevation sites remained abiotically unfavorable for cheatgrass.

## DISCUSSION

We developed a new modeling approach to improve understanding of an important climate–species relationship and investigate how climate change has influenced the potential distribution of cheatgrass over the last 30 years (1989–2019). Our results indicate that modeling the distribution of an invasive annual grass species using microclimate covariates linked directly to germination is a viable method to understand the relationship between climate and potential distribution. The combination of site‐level soil‐microclimate data and information about germination response allowed our model to identify soil conditions in space and time that favor germination and result in persistent cheatgrass presence. The relationships derived from our model also allowed us to track changes in habitat suitability for cheatgrass across years.

### Comparison to remotely sensed models

Our model predicted the distribution of cheatgrass well, with accuracy (72%) similar to previous species distribution models despite using only four microclimate‐based covariates. Cheatgrass‐specific distribution models based on remotely sensed covariates with a similar geographic range had similar performance: Downs et al. (2016) reported an overall accuracy of 71%, Bradley and Mustard (2006) reported an overall accuracy of 61%, and Bradley et al. (2018) reported an overall accuracy of 74%. The advantage of our approach is that it provides more direct inference about the relationship between climate and cheatgrass distribution and abundance. Our model is also able to capture interannual variation in climate suitability, whereas previous models that correlate average climate to distribution based on several years of reflectance data (Bradley, 2009) could not predict variation among years.

Our model was unable to explain the variability in cheatgrass cover as well as remotely sensed models. Our model predicted percent cover with an *R* ^2^ value of 0.22, whereas other remotely sensed models produced more accurate estimates of percent cheatgrass cover: Bradley et al. (2018) reported an *R* ^2^ value of 0.32 (3769 testing points), Peterson (2005) reported an *R* ^2^ of 0.5 (75 testing points), and Peterson (2006) reported an *R* ^2^ of 0.24 (806 testing points). More specifically, our model failed to predict high values of cheatgrass cover, which is a similar problem experienced by these other remote sensing models. The inaccuracy of our abundance model probably reflects the importance of many non‐climate factors, such as disturbance and competition from native vegetation, in determining cheatgrass abundance.

### Species–climate relationship

Our study indicates that areas characterized by warm and wet fall seasons (October–December) and warm and wet springs (March) have the greatest abiotic potential for cheatgrass presence and abundance. Fall conditions had a stronger positive effect on cheatgrass presence and abundance than spring conditions (Figure 5). Previous studies have indicated hot, dry summer atmospheric conditions as a key factor in cheatgrass dynamics (Bansal & Sheley, 2016; Bradley, 2009; Brummer et al., 2016) and strong topographical effects of elevation and aspect, but our initial screening of covariates indicated that fall and spring soil conditions were more informative to our model than summer soil metrics. Though summer conditions are likely to affect annual species indirectly by shaping competing perennial vegetation (Condon et al., 2011), summer conditions should not have large effects on cheatgrass seeds that largely remain dormant until germinating in the fall or early spring (Hulbert, 1955; Mack & Pyke, 1984). We suspect that the impacts of summer climate found in previous models reflect their correlation with elevation, seasonal soil moisture timing, and shifts in vegetation type. Experimental findings from field studies indicate that year‐round warming has a positive effect on cheatgrass (Blumenthal et al., 2016; Compagnoni & Adler, 2014), whereas late spring and summer warming alone had a negative effect on cheatgrass cover and fecundity (Larson et al., 2017). Year‐round warming would increase the quantity of warm, wet conditions in the spring and fall, which is shown by our model to increase suitability for cheatgrass.

Our results provide insight into the climate factors that generate increasing resistance to cheatgrass invasion with increasing elevation. Current hypotheses link resistance to water availability, soil temperature, and competition (Chambers et al., 2019; Chambers, Bradley, et al., 2014; Chambers, Miller, et al., 2014), but disentangling the role of these abiotic factors is difficult because they are tightly correlated. Our results support the conclusions of Roundy et al. (2018) that resistance to cheatgrass invasion depends on spring and fall soil conditions, with colder fall and spring soils, and increasing elevation, reducing the abiotic potential for cheatgrass establishment (Figure 6; Appendix S1: Figure S2). The explanatory power of our model, with explicit ties to germination, indicate further that fall and spring soil conditions are important due to their influence on germination. We also anticipate that fall and spring soil conditions are linked to cheatgrass invasion, because they may allow cumulative periods of growth, captured by the summing nature of our microclimate metric, that can be utilized by a winter annual grass for early growth.

Our models generally indicate that warmer and wetter soil in the fall and spring periods, in both space and time, increase the probability of cheatgrass presence, and to a lesser degree, cheatgrass cover (Figure 5). However, the effects of temporal anomalies in spring conditions did not follow this pattern and indicated that warmer and wetter conditions decreased cheatgrass presence (Figure 5). We hypothesize that this discrepancy is indicative of the complex relationships between spring soil microclimate and cheatgrass dynamics. Though warm and wet spring soils have been shown to be beneficial to cheatgrass, they also decrease the likelihood of fire (Pilliod et al., 2017), a factor strongly linked to cheatgrass distribution (Bradley et al., 2018). Without accounting for fire or spatial factors that determine the abundance of competing native flora, our model predicted anomalies in spring soil microclimate to be the least informative parameter in both our abundance and distribution models. We anticipate that accounting for interactive effects of spatial factors of fire and native species composition with spring soil conditions would not only improve model fit but would also indicate a strong positive effect of warm and wet spring soils when native plant cover is low. This would support findings from Bradford and Lauenroth (2006) showing that the effect of temporal conditions only becomes important in scenarios where spatial factors such as total plant cover and disturbance history allow a sizable response to interannual variation in weather.

The contrasting accuracy of our distribution and abundance models suggests that different factors control cheatgrass distribution and abundance. Our model is based on the abiotic factors that directly influence germination. The success of this model in explaining cheatgrass presence/absence indicates a primary role for germination and periods of wet and warm shallow soil microclimate. In contrast, the low explanatory power of our model for abundance indicates the importance of other biotic and abiotic factors likely unrelated to germination and shallow soil microclimate. This fits well with the conclusion of Bradford and Lauenroth (2006) that climate drives susceptibility to annual grass invasion, and disturbance regime dictates the severity of invasion. There are many studies that indicate the positive impact of disturbance, especially fire, on annual grass abundance (Bradley et al., 2018; Condon et al., 2011; D'Antonio & Vitousek, 1992; Fusco et al., 2019; Gill et al., 2018; Williamson et al., 2020). Failure to account for disturbance history or competitive interactions may limit our model's ability to distinguish between high and low cheatgrass cover, primarily because cover of competitive species and lack of disturbance may limit propagule pressure and thus complicate species response of annual plants to favorable soil conditions.

### Trends in cheatgrass distribution

Our analysis suggests that climate change has already benefitted cheatgrass and expanded its potential range 10%–17% across low and mid‐elevation sites (Figure 6). These results are consistent with trends found in remotely sensed data by Smith et al. (2022), showing an increase in annual grass dominance across sites with elevation <2100 m, and Pastick et al. (2021), who found similar increases in cheatgrass distribution and cover across low and mid‐elevation sites. Understanding new changes in distribution is critical due to potential positive feedback where even small amounts of cheatgrass (<10% cover) have been linked with heightened wildfire risk (Pastick et al., 2021), which can quickly lead to post‐wildfire dominance of invasive annual grasses and more subsequent fires (Bradley et al., 2018; D'Antonio & Vitousek, 1992).

### Benefits of our approach

Our approach only considers soil moisture and temperature metrics known to directly influence cheatgrass germination. This sets up a relatively simple model with few covariates to describe a site's potential for cheatgrass compared with current remotely sensed models (Bradley et al., 2018; Pastick et al., 2021). Using a model with fewer covariates also simplifies interpretation. Because our approach explains field observations of cheatgrass distribution and abundance solely as a function of microclimate covariates, we are able to understand climate constraints and preferences of cheatgrass without introducing additional uncertainty that occurs when estimating cheatgrass distribution based on remotely sensed imagery. In addition, our model can explain interannual variation in cheatgrass suitability at a single site, in contrast to models limited to inference of mean climate conditions at each site.

Our results may be useful for natural resource management as it indicates not only which are the locations that may be vulnerable to cheatgrass invasion but also provides the tools to understand which new areas may become vulnerable with current trajectories of climate that alter climatic suitability for cheatgrass presence. This allows managers to not only mitigate activities in locations that have recently become vulnerable to cheatgrass invasion but also provides time for management to prepare for future invasion vulnerability.

### Potential limitations of our approach

The soil microclimate approach we used is appropriate for systems where water availability is a key limiting factor but may not provide meaningful information regarding a species' potential in systems where resource availability or survival are not explicitly tied to soil moisture and soil temperature metrics. Our study also focuses on a species with an annual life history, meaning that annual germination and growth favorability metrics are very relevant to each year's distribution and abundance. Perennial species could be less sensitive to factors regulating germination and seedling performance. We suspect that the distribution and abundance of perennial species will have different microclimate requirements with lag effects of favorable or unfavorable conditions being important. Finally, our approach depends on lab trials to generate germination curves, and thus requires more resources than remote sensing approaches to map distribution, though it remains unknown whether rate sum values from one species are adequate estimates of soil favorability for other species. Currently, it is best suited for understanding climate–species relationships or predicting susceptibility to invasion.

Our model indicates whether cheatgrass persistence may be possible due to relationship with climate but does not include disturbance, a major driving factor behind exotic annual grass dominance (Bradley et al., 2018; Fusco et al., 2019; Pastick et al., 2021). While our approach illustrates that the influence of climate alone may determine the distribution and persistence of cheatgrass, we encourage future effort to study how soil favorability metrics interact with disturbance and competitive native plant abundance to better understand how/where climate and disturbance may interact to create systems dominated by exotic annual grasses, where impacts of invasion are most severe.

## AUTHOR CONTRIBUTIONS

Tyson J. Terry and Stuart P. Hardegree conceived the study. Tyson J. Terry and Peter B. Adler developed and ran statistical models. Tyson J. Terry and Stuart P. Hardegree developed and ran germination models. Tyson J. Terry led writing with comments and multiple rounds of feedback from all co‐authors.

## CONFLICT OF INTEREST STATEMENT

The authors declare no conflicts of interest.

## Supporting information


Appendix S1:

## Data Availability

Data and code (Terry, 2023) are available in the Open Science Framework repository at https://doi.org/10.17605/OSF.IO/7PUVN.
