# Supplementary material for: Modeling cheatgrass distribution, abundance, and response to climate change as a function of soil microclimate
Source: Ecol Appl. 2024 Sep 16;34(8):e3028. doi: 10.1002/eap.3028 (PMC11610693; doi:10.1002/eap.3028)
Supplement: Supplementary file 1 — Appendix S1: [file EAP-34-e3028-s001.pdf]

Journal: Ecological Applications

Manuscript Type: Article

Title: **Modeling cheatgrass distribution, abundance, and response to climate change as a function of soil microclimate**

Tyson J. Terry, Stuart P. Hardegree, Peter B. Adler

Supplemental Tables

Table S1. Data sources and information on field observations used for model training and testing.

Source	# Points	Spatial extent	Sampling Years
Bradley and Mustard 2005	28	6 x 30m transects	2003-2004
Brummer et al. 2016	275	20 20x50 cm frames in 1 ha	2009-2015
Fleishman 2015a, b	598	3 x 50m transects	2006-2015
Vegetation measurements in bird habitat, Erica Fleishman unpublished	108	3 x 50m transects	2016
Field Surveys, Erica Fleishman unpublished	89	3 x 50m transects	2016
Field Surveys, Adam Mahood unpublished	7	6 x 50 m transects	2016
Nevada Department of Wildlife Surveys	1177	3 x 50m transects	2011-2016
Peterson 2005	184	.1 ha ocular estimates	2002-2003
Sage Step control plots McIver et al. 2014	185	3 x 30m transects	2006-2007

Table S2. Statistical summary of the GAM Presence/Absence Model.

**Presence/Absence (Distribution) Model**				
Parametric coefficients:				
	Estimate	Std. Error	T value	Pr(>|t|)
(Intercept)	0.14	0.04865	2.887	0.0039
Approximate significance of smooth terms:				
	edf	Ref.df	Chi.sq	P-value
s(Preceding Fall Rate Sum)	6.316	9	94.73	<2e-16
s(Preceding March Rate Sum)	5.666	9	42.84	<2e-16
s(Average Fall Rate Sum)	4.095	9	32.76	<2e-16
s(Average march Rate Sum)	6.314	9	35.72	<2e-16

Table S3. Statistical summary of the GAM Cover Model.

**Cover (Abundance) Model**				
Parametric coefficients:				
	Estimate	Std. Error	T value	Pr(>|t|)
(Intercept)	1.71832	0.03089	55.63	<2e-16
Approximate significance of smooth terms:				
	edf	Ref.df	F value	P-value
s(Preceding Fall Rate Sum)	4.361	9	13.282	<2e-16
s(Preceding March Rate Sum)	7.2	9	11.082	<2e-16
s(Average Fall Rate Sum)	4.443	9	4.863	<2e-16
s(Average march Rate Sum)	7.342	9	7.251	<2e-16

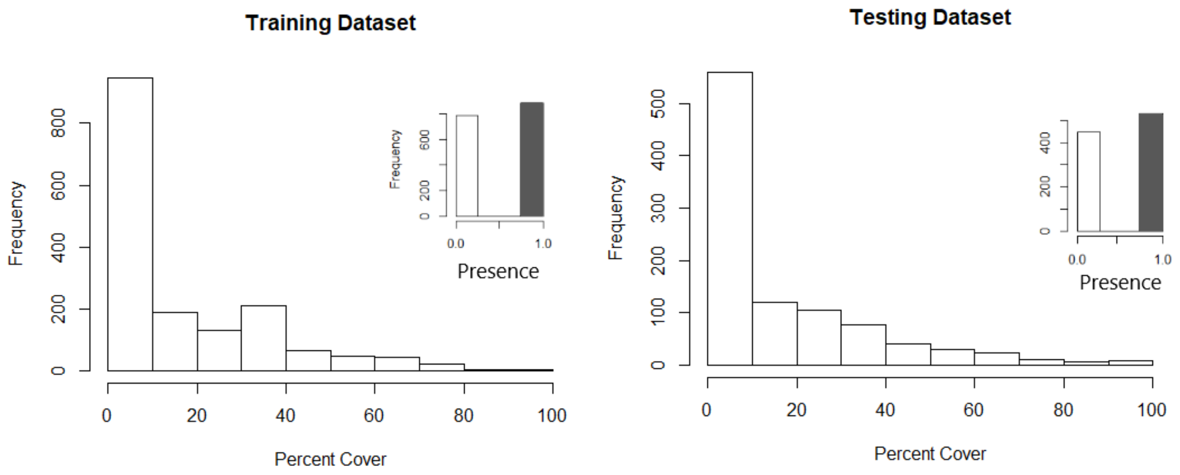

Figure S1. Histograms showing the distribution of cheatgrass cover data across study sites for our training (left) and testing (right) datasets. Insets display presence absence ratios, with the dark bar representing locations where cheatgrass was observed with >2% herbaceous cover (present).

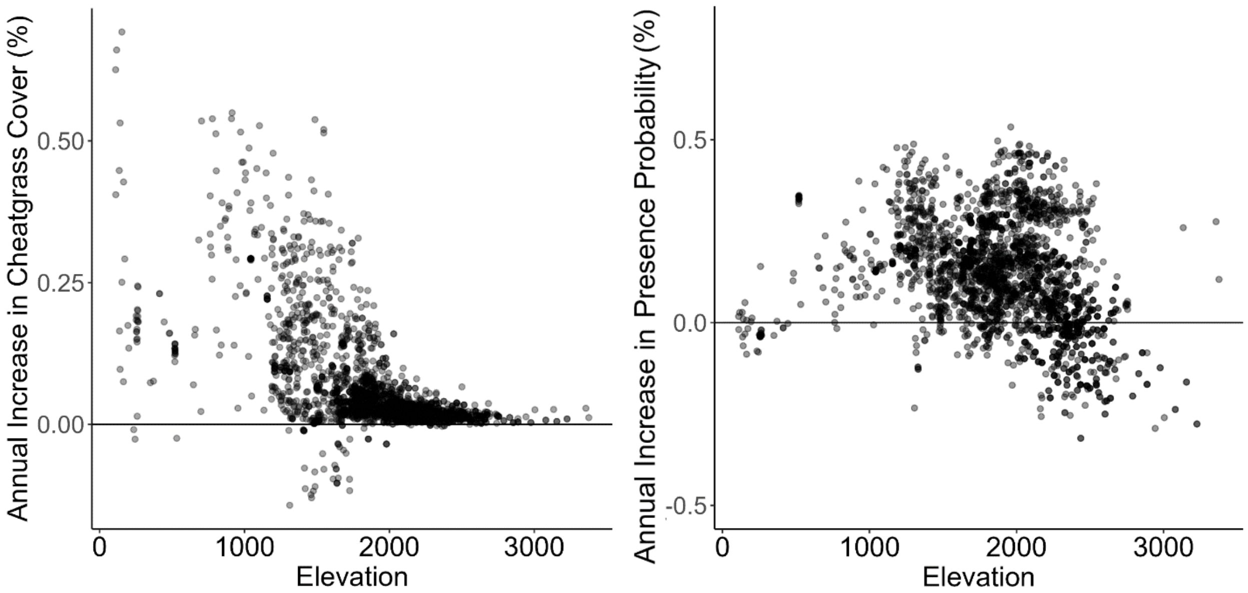

Figure S2 Estimated annual increase in cheatgrass cover (left) and probability of cheatgrass presence (right) due solely to changes in climate across elevation at study sites (n=2662). Each point represents the average annual change in predicted cheatgrass cover for each site (left) or average change in predicted probability of cheatgrass presence (right). All estimates are based on annual microclimate conditions during the period 1989-2019.
